# Guest Editorial Special Issue on Time-Sensitive Networks for Unmanned Aircraft Systems

**DOI:** 10.3390/s21186132

**Published:** 2021-09-13

**Authors:** Hwangnam Kim, Yong Wun Jung, Honghai Zhang

**Affiliations:** 1 School of Electrical Engineering, Korea University, Seoul 02841, Korea; 2 Unmanned Vehicle Advanced Research Center, Korea Aerospace Research Institute, Daejeon 34133, Korea; jyw@kari.re.kr; 3 Platform and System, Google Inc., Kirkland, WA 98033, USA; honghaiz@google.com

**Keywords:** low latency, time-sensitive network, deterministic network, cyberphysical systems

## Abstract

In this special issue, we explored swarming, network management, routing for multipath, communications, service applications, detection and identification, computation offloading, and cellular network-based control in time-sensitive networks of unmanned aircraft systems.

Unmanned aircraft systems (UASs), commonly known as drones, have been increasingly investigated in various research and service areas to support humans or enhance their operations. Drones can be applied to a wide range of services, e.g., surveillance, patrols, platooning, vehicle traffic control, search-and-rescue missions, wild fire monitoring, product delivery, or video taking. In the course of performing the missions, UASs can construct a flying ad hoc network with their neighbors, send their flight status and other information sensed through a variety of sensors and cameras to a ground control station (GCS), and receive flight or mission commands from it over the network, most of which should be carried out in a timely manner. In particular, lost or delayed flight control commands can lead to diverse accidents and sometimes catastrophes. Therefore, participating UASs require a time-sensitive network that should guarantee deterministic properties in packet delivery, positioning, navigation, and synchronization. Note that time-sensitive networking (TSN) is a suite of standards for time synchronization, scheduling and traffic shaping, path reservations and selection, and fault-tolerance, which is under development by the Time-Sensitive Networking task group of the IEEE 802.1 working group.

There are many challenges in this research area that need to be solved or improved to implement time-sensitive network for UASs. The special issue “Time-Sensitive Networks for Unmanned Aircraft Systems” of Sensors journal have solicited paper submissions concerning the research findings that explored new methods in order to contribute in various aspects of time-sensitive networks for unmanned aircraft systems, which are communications, network control and management, multi-path routing, swarming, applications, detection and identification, computation offloading while manging UASs, and cellular system based control.

This Special Issue covers both the theoretical and practical aspects of time-sensitive networks for UASs. With many submissions from different corners of the world, the responses to our call for papers for this Special Issue were quite satisfactory. During the review process, each paper was assigned to and reviewed by at least three experts who are eminent researchers in the same relevant areas as the papers, and has undergone up to three rigorous rounds of the peer-review process. Among the dozens of papers received for this Special Issue, only ten high-quality papers covering various aspects of time-sensitive networks for UASs were selected for this Special Issue. The papers selected for this Special Issue represent the quality, breadth and depth of the field of applying time-sensitive network technologies and their relevant technologies to UASs.

Thanks to the highly efficient administrative support from MDPI’s *Sensors* journal, we were able to accept these high-quality papers covering various aspects of time-sensitive networks for UASs. In addition, we thank all authors for their valuable collaboration and contributions to this Special Issue and we also would like to thank the international team of editorial staffs for their highly efficient administrative support and reviewers for their thoughtful and constructive criticisms in evaluating papers.

## Summary of the Special Issue

In operating multiple UAVs, each UAV is supposed to carry out its own mission while collaborating with the other UAVs. Therefore, we need to manage connectivity between any pair of UAVs while not disrupting the mission of each UAV. L. M.B. Ribeiro et al. [1] presented an interface manager (IM) that can improve communication in multi-UAV networks in order to improve the stability of link quality of UAV-to-UAV (U2U) communication in the context that the distance between each other UAV varies as individual trajectories and the medium dynamics cannot be accurately estimated. The proposed interface manager is expected to define the best interface for sending messages based on on-flight state dynamically detected and estimated from the wireless medium.

In order to make UAVs configurable according to a given mission, it is necessary to guarantee inter-working with their additional installed hardware and software components. A Robot Operating System (ROS) is usually employed as a middle-ware to support collaboration among components in a single robot or a collection of robots, and it is also employed to construct communication paths between components within UAVs. This is because an ROS provides to those components several services necessary for inter-working, such as hardware abstraction, low-level control for device, and inter-process message passing for operations. Observing it might lead to security vulnerabilities through various aspects to append additional hardware and software through ROSs to enhance operations with unmanned aerial vehicles (UAVs); H. Lee et al. [2] proposed a security framework to improve the security of an UAS. The proposed framework works in the robot operating system (ROS) and deals with security issues essential for flight missions. The authors showed, based on real-time experiments, that the proposed framework could be effective in preventing cyber attacks attempting to use the vulnerability of the ROS.

Any type of connectivity between a control point (operator or ground control station) and its peer UAV(s) should be kept reliably and consistently in order to prevent any kind of accidents caused by loss or delay of control. The following papers [3,4] present how to address communication instability when the wireless medium that is common means to connect operators and their UAVs become unreliable.

In a wireless environment, communication is exposed to risk of packet loss or delay as previously mentioned; then, subsequent retransmissions inevitably occur, and the resulting delay becomes fatal to UAV control and operation. Thus, it is strongly required that control packet loss and delay should be prevented. In order to stably control a UAV by transmitting control messages in a predetermined manner, W. Lee et al. [3] proposed a control packet transmission scheme, ConClone. ConClone replicates packets according to analytical models that they proposed and then transmits them over multiple network connections to increase the probability of successful control packet transmission. The authors explained in detail how to implement the proposed packet transmission scheme and showed its performance results with theoretical analysis and experiments. In particular, they showed that equipping multiple network interfaces in a UAV does not make the UAV unstable in terms of hardware balance, and also they conducted rigorous channel model analysis for both air-to-ground (A2G) and air-to-air (A2A). Furthermore, W. Lee [4] devised the MuTran to enhance the work in [3]. The ConClone in [3] basically duplicates control packets to increase delivery ratio of control packets, but control packets could be interfered with data packets since those types of packets are inserted into the same queue. This observation motivated the author to consider the packet type and thus decide to maintain an independent queue for control packets separated from the other queue for data packets. Based on this separation, the author could improve the inter-packet transmission delay of control packets as well as reliability of control packet transmissions.

From the studies [3,4], we learn the following lessons. Since any networking problems or wireless communications could lead to control packets being lost or delayed, the unstable drone control cannot be avoided when they become worse, and consequently operators cannot operate UAVs and deal with any flying problem in real time. Therefore, in order to construct time-sensitive networks for UAVs, it should be carried out at the first hand to make all the connections within a fleet of UAVs reliable and stable.

There are many studies on UAV swarms to overcome the limitations of a single UAV’s capabilities in terms of payload, detection range, and hardware capability. In order to control a collective behavior and conduct self-organization control of a swarm of 10 UAVs divided into two clusters of five agents, Z.A. Ali et al. [5] proposed a hybrid meta-heuristic algorithm that merges the particle swarm optimization (PSO) with the multi-agent system (MAS). In the algorithm, PSO determines the best agents of a given cluster, and MAS chooses the best agent as the leader of each cluster. The leader is supposed to find the optimal path for each cluster, and the clusters are organized into a formation by implementing the PSO with the MAS model, which coordinates the agents inside the cluster. Based on the simulation studies, the authors showed that the proposed meta-heuristic algorithm could be effective for both inside the clusters and the greater swarms according to the mission requirements in the comparison with the existing NSGA-II. The authors also showed that any number of clusters with any number of UAVs could be compatible with the proposed algorithms.

As a specific application of UAVs, UAV-based delivery systems such as Amazon Prime are receiving a lot of attention. In such a system, a management plan is needed to optimize the UAV delivery route in real time. S. Kim et al. [6] proposed a UAV routing algorithm to find the optimal round-trip route for a UAV that delivers goods from a warehouse to a customer. The algorithm determines the optimal route by minimizing the transportation distance when the maximum range and loading capacity of the UAV are given. In the course of optimal routing, the proposed algorithm observes service indicator variables in the objective function to optimize the number of UAVs in service per base, so that it could minimize total fixed and variable costs. It also finds the optimal path to the UAV by eliminating the sub-tour and optimally allocating the UAV. In addition, the authors showed that the algorithm can be used to operate multiple UAVs at the same time to meet the demands of one customer.

It is difficult to identify UAVs since they fly around at low altitudes and with small radar cross sections (LCS); hence, a new identification scheme for UAVs should be devised to overcome the limitation of the existing system. D. Park et al. [7] present a method to employ a deep learning based classification algorithm based on micro-Doppler signature of UAVs represented on radar spectrum images. The proposed scheme was designed to detect and identify UAVs in real time. Specifically, UAV signals and human activity signals were recorded in various scenarios using the FMCW radar, and radar spectrogram datasets were generated with high diversity through STFT, data refinement, and data augmentation methods. Then, the characteristics of the radar spectrogram dataset using ResNet-18 were analyzed to confirm the optimal data form and model structure. Finally, the authors designed the REsNet-SP model, which has been built in the way of compressing and stabilizing the existing ResNet-18 and thus was more suitable for real-time systems, and then proved that the resulting model has high stability, accuracy, and faster computation time than the ResNet-18 model.

Based on the observation that the influence of UAVs’ mobility on time-related performance should be precisely investigated to accurately realize time-sensitivity networks over multiple UAVs, S. Park et al. [8] proposed a new co-simulation scheme for multiple UAV networks, which executes both the flight simulation and the network simulation at the same time. The simulation scheme can analyze the dependency between the flight and network status and concentrate on keeping the consistency of simulation state through synchronization among simulation components. The simulation scheme has been designed to run the existing simulators, which are a flight simulator and a network simulator, combine the information of them to efficiently resolve the dependencies between the flight and the network simulators while simulating the multiple UAVs’ network, and employ the HLA time management model to synchronize the two simulators. The scheme has been implemented and verified to simulate multiple use cases and generate performance evaluation results. The authors showed that the proposed simulation scheme can be used for any pair of flight and network simulation as far as the application programming interface is correctly specified.

Due to the UAV’s scarce resources, all the tasks required to perform the mission of the UAV cannot be executed within the UAV. If so, any task cannot accomplish its own mission; in particular, the time-sensitive task of controlling the position or attitude of UAV can be easily interfered with by other tasks, which can incur catastrophic accidents. A. Koubaa et al. [9] proposed a system architecture of computation offloading for drones connected to the Internet. This architecture was motivated by the development of applications where the UAV video is streamed into the cloud and the cloud uses a deep learning system to analyze the video data in real time for end-users. The authors showed, via empirical studies, that the proposed offloading system could reduce the energy consumption of UAVs while performing computer vision applications using CNN algorithms, which is known as a computation-oriented task, in real time.

Cellular systems can be another important infrastructure that connects UAVs to their operators, which is different from existing infrastructure such as direct mode or ad hoc mode since cellular systems directly support time-sensitive property to establish connections for time critical applications. It is therefore necessary to examine various communication modes allowed in cellular systems and employ the mode that is appropriate for UAV operations. M. Han et al. [10] proposed a dynamic bandwidth part (BWP) allocation scheme that switches between two multiplexing methods, dynamic multiplexing (DM) and orthogonal slicing (OS), so as to minimize an impact of ultra-reliable low latency communication (uRLLC) traffic on enhanced Mobile Broadband (eMBB) traffic. Considering uRLLC traffic is well-suited for UAV control and operation, the traffic can be exploited to construct a communication path for UAV control and mission control systems.

## Data Availability

Not applicable.
